# Modeling and Experimental Study of the Localized Electrochemical Micro Additive Manufacturing Technology Based on the FluidFM

**DOI:** 10.3390/ma13122783

**Published:** 2020-06-19

**Authors:** Wanfei Ren, Jinkai Xu, Zhongxu Lian, Peng Yu, Huadong Yu

**Affiliations:** Ministry of Education Key Laboratory for Cross-Scale Micro and Nano Manufacturing, Department of Mechanical and Electrical Engineering, Changchun University of Science and Technology, Changchun 130012, China; renwanfei2020@163.com (W.R.); lianzhongxv@126.com (Z.L.); 13620788904@163.com (P.Y.)

**Keywords:** localized electrochemical, micro additive manufacturing, 3D micro overhang structure, FluidFM

## Abstract

In this work, the localized electrochemical micro additive manufacturing technology based on the FluidFM (fluidic force microscope) has been introduced to fabricate micro three-dimensional overhang metal structures at sub-micron resolution. It breaks through the localized deposition previously achieved by micro-anode precision movement, and the micro-injection of the electrolyte is achieved in a stable electric field distribution. The structure of electrochemical facilities has been designed and optimized. More importantly, the local electrochemical deposition process has been analyzed with positive source diffusion, and the mathematical modeling has been revealed in the particle conversion process. A mathematical model is proposed for the species flux under the action of pulsed pressure in an innovatively localized liquid feeding process. Besides, the linear structure, bulk structure, complex structure, and large-area structure of the additive manufacturing are analyzed separately. The experimental diameter of the deposited cylinder structure is linearly fitted. The aspect ratio of the structure is greater than 20, the surface roughness value is between 0.1–0.2 μm at the surface of bulk structures, and the abilities are verified for deposition of overhang, hollow complex structures. Moreover, this work verifies the feasibility of 3D overhang array submicron structure additive manufacturing, with the application of pulsed pressure. Furthermore, this technology opens new avenues for the direct fabrication of nano circuit interconnection, tiny sensors, and micro antennas.

## 1. Introduction

As micro metal structural components are increasingly applied in the fields of aerospace [1], microelectronic packaging [2], micro-sensors [3], bio-medical [4,5,6,7], dissipating heat [8], and anti-reflection [9], nowadays, many processing technologies have emerged for the manufacture of metal microstructures, including precision milling [10], elliptical vibration cutting [11], laser etching [12], electrical discharge cutting [13] and other ultra-precision processing technologies. However, the additive manufacturing (AM) method [14] of fabricating micro metal components has been greatly developed, due to its advantages of low consumption, environmental protection and positive design. For the micro three-dimensional (3D) structures, u-SLS technology has a high printing rate, while its printing accuracy is less than 5 μm [15]. Direct ink writing (DIW) [16], and electro-hydraulic jet printing [17] have a high resolution, but they cannot directly produce such overhanging structures, and post-treatment annealing is required instead. By focused electron beam induced deposition (FEBID) [18], small-scale printing can be realized, but it requires an inert gas or vacuum environment during processing. Although the resolution and manufacturing accuracy are very high in local oxidation nanolithography, their deposition rate is extremely slow [19,20]. Additionally, the micro stereolithography (MSL) technology [21], direct electron beam writing (EBW) [22] and two-photon absorption (TPA) [23] for non-metal printing additive manufacturing technology have high printing accuracy. These technologies, however, are only suitable for polymer printing, and subsequent treatments are required when fabricating metal microstructures. Actually, metallization can be achieved by post-coating or deposition, but it is so hard to form micro metal structures in one step, and to properly control the source of post-processing cumulative errors. Localized electrochemical micro-additive manufacturing technology based on the FluidFM probes is inspired by the localized electrochemical deposition (LECD) technology. As a matter of fact, LECD technology has evolved into a number of hybrid variants, after more than two decades of development. For example, the LECD process is also known as electrochemical additive manufacturing (ECAM) [24,25,26,27,28] and the electrochemical printing (EcP) [29] is also the maskless electrodeposition process which can deposit metal and alloy structures on conductive substrates. Another technology named meniscus-confined electrodeposition (MCED) uses the thermodynamically stable behavior of microscale liquid meniscus to construct the 3D structures and fabricate complex metal microstructures [30]. As shown in Table 1, this work summarizes the comparison of the above technologies, in terms of deposition rate, minimum deposition diameter, high aspect ratio, complex structure, formation quality of surface, motor resolution dependence and subsequent processing, etc.

As one kind of electrochemical micro-additive manufacturing process, the localized electrochemical micro-additive manufacturing technology based on the FluidFM (LECD-FluidFM) applies a bias voltage between the cathode and anode, which are both immersed in an acid electrolyte solution, and hence causes the metal ions salt solution to flow from the FluidFM probe via air pressure, as a result metal ions are reduced to metal atoms, which adhere to the substrate, forming a 3D structure. This technology can directly print micro-metal overhang structures at sub-micron resolution without masks or support materials. Besides, neither strict vacuum environment nor inert gas is required during printing, and it is also not necessary to introduce external high-density thermal energy input, as compared to the normal metal 3D printing methods. Furthermore, it does not generate thermal residual stress while depositing. LECD-FluidFM can overcome the disadvantage of the lower deposition rate and the fact that only simple structures can be printed. Meanwhile, two function roles of a special AFM (atomic force microscope) probe were demonstrated: being a microscope probe, and a species transportation tool [31,32]. More significantly, in 2019, Giorgio et al. [33,34] described local distribution of metal ions in a standard three-electrode electrochemical cell for metal microstructure AM. Furthermore, lots of experiments were conducted to obtain basic data, and the diameter of the deposit could be changed dynamically by adjusting the applied pressure. However, research on the mathematical model based on the LECD-FluidFM is still rare. Only a few scholars have done fundamental research on electrodeposition models. Recent research focused on the establishment of a current density model, based on the Fick diffusion law and Butler-Volmer equation electrode kinematics in electrochemical deposition systems [35,36]. Additionally, Volgin et al. [37] established a mathematical model to form 3D microstructures, using LECD based on a moving disk microanode, and then the relationship between the electrodeposition parameters and the shape of the microstructure were determined.

The above literatures summarized the process of forming metal microstructures. However, these methods have some limitation on fabrication of micro complex metal overhang structures. In the mathematical modeling method, the role of gas pressure in metal forming mechanisms was not fully described. The other parts of this paper include the construction of experimental facilities, the mathematical models behind the forming mechanism, and experimental research and performance characterization.

## 2. Experimental Section

The LECD technology owes much to the development of electrode fabrication technology. Previously, the electrodes were almost solid metal. In order to improve the locality of deposition, the diameter of electrodes was sharply decreased. However, researchers found it difficult to obtain an expected diameter, so they began to focus on the hollow capillary electrode. The capillary was manufactured with a tiny hole, and then evolved from a single hole into two or several holes. However, even so, the technology based on the electrode or capillary highly relies on the positioning and repeated positioning accuracy of the anode moving motor. Referring to the detection method of AFM technology, the hollowing of the AFM probe was introduced to the LECD technology. The metal salt solution passed through the cantilever channel to the tip of AFM for localized liquid feeding via pressure control. By accurately detecting the atomic force, the AFM cantilever could convert force signal to electrical signal and give feedback to the control system.

### 2.1. Experimental Equipment

**Figure 1:**
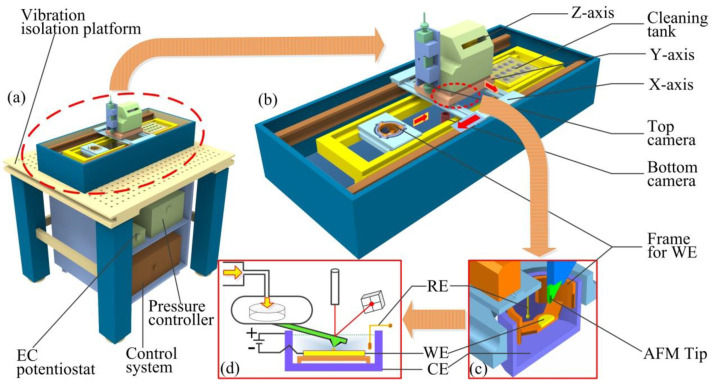
Schematic model of the localized electrochemical deposition based on the hollow AFM probe. (**a**) The whole view of the electrochemical deposition system. As a brain of the whole system, the control system decides where and what shape to print and how big to manufacture, and controls all the subsystems. The vibration isolation platform is the base for the whole printing system. The EC (electrochemical) potentiostat maintains a stable potential difference between the RE (quasi-reference electrode) and WE (working electrode). The pressure controller is connected to the end of the AFM cantilever. The amplitude of the pressure can control the volume throughput from the tip of the FluidFM. (**b**) The enlarged view of the printer displaying the key components in more detail. The X-axis and Y-axis represent the same level of precision having a repeat positioning accuracy of +/− 250 nm. The Z axis has high precision in repeat positioning up to 5nm. The cleaning tank is used for cleaning the AFM tip during the electrodeposition process. Moreover, the top and bottom cameras are used to monitor the manufacturing and assembly processes. (**c**) The cross-section of the printing chamber. When it is working all the electrodes are emerged under the acid solution. RE denotes the reference electrode, CE (counter electrode) is the counter electrode connecting the positive pore of the power, and WE is the working electrode linking to the negative pore of the power. The AFM tip is the key part of the whole technology because the metal salt solution comes from the hollow AFM probe. The frame of the WE is also important for insulation of three electrodes in electrodeposition process. (**d**) The schematic diagram of the localized electrochemical deposition based on the hollow AFM probe.

As shown in the Figure 1, the whole equipment is composed of the XYZ coordinate system in both macro/micro scale, FluidFM printing system, electrolyte unit, cleaning unit, pressure control system and positive vibration isolation unit. The minimum positioning accuracy of the bidirectional *X*/*Y*-axis nano mobile system is less than 500 nm, and the positioning accuracy of the *Z*-axis nano mobile system is less than 5 nm. When establishing the FluidFM-based printing system, the AFM cantilever was hollowed out, and FIB was used to process a micro-hole at the tip of the AFM probe. In this experiment, the probe with a diameter of 300 nm (the minimum size can be 50 nm) was selected. The metal salt solution reservoir was connected to the rear end of the cantilever. The metal salt solution flowed out of the FluidFM tip through the hollow cantilever under the action of air pressure. The cleaning unit was used to adjust the position of the probe and control the signal feedback of the AFM probe cantilever. Furthermore, there were several solution tanks in the cleaning unit, which were filled with different buffer solutions for cleaning the FluidFM probe tip online, so as to prevent the tip from getting clogged ensuring a longer service life during the electrodeposition process. The pressure control system was designed to control the liquid supply in a localized and quantitative manner. The pressure control system was connected to the end of the AFM cantilever of the printing system through a thin gas tube, and realized the localized outflow of the metal salt solution via software collaborative control, thereby achieving the localized electrochemical deposition of the metal. For the active vibration isolation unit, the entire printing device was mounted on an air floating vibration isolation platform, and the four feet of the air floating platform could provide air sources for achieving active vibration isolation.

### 2.2. Experimental Parameters

To further introduce this technology, a high-precision localized electrochemical deposition experiment of copper metal was carried out. Copper is an excellent material for lossless information transmission in antennas, wires, microchips, signal transmission, and radio wave propagation. Only by satisfying the above basic theory of the material forming process model can the precise manufacture of tiny and complex copper structures be realized. The effectiveness of the entire model is described by the precision and complexity of the microstructure formed by electrochemical micro-additives. The parameters used in the experiment are shown in Table 2. For the solution aspect, the copper solution is mixing 0.5 M copper sulphate solution in sulfuric acid (51 mM) and hydrochloric acid (0.48 mM). The supporting solution is mixed by H_2_SO_4_ solution (54 mM) and HCl solution (0.5 mM). For the parameter aspect, the voltage applied to the cathode substrate is −0.52 V. The approaching voltage of FluidFM is set at 50 mV. Additionally, the pressure is set at 20 mbar.

### 2.3. Characterizations

In order to better understand the deposition mechanism of highly localized electrochemical deposition based on the FluidFM, various methods have been used to characterize the deposition process and the composition and morphology of the deposits. High-magnification top-view and bottom-view cameras were used to monitor the deposition process. The size of parts manufactured by electrochemical micro-additives is small, so it is difficult to measure the roughness of the surface with a contact surface roughness measuring instrument. The 3D structures were manufactured by localized electrochemical deposition and further characterized by lots of advanced instruments. In this work, the surface roughness value was measured by a white light interferometer (Zygo corporation, NewView 8000, Berwyn, PA, USA), and the features of 3D structures morphology and chemical analysis (EDS) were characterized using scanning electron microscope (SEM) (ZEISS, EVO 20, Jena, Germany). Besides, height detail was captured by a laser scanning confocal microscope (LSM) (ZEISS, LSM700, Jena, Germany).

## 3. Results and Discussion

This work evaluates the overall performance of LECD-FluidFM technology from four dimensions: wire structure, volume structure, complex structure and large-area structure. The experimental verification and analysis investigated the high aspect ratio, print reproducibility, surface roughness, and micro-morphology of the deposits of tiny metal structures. Furthermore, three-dimensional complex structures and large-area structures were verified and explored, and the component content on the surface of the structure was analyzed.

### 3.1. Wire Structure

**Figure 2:**
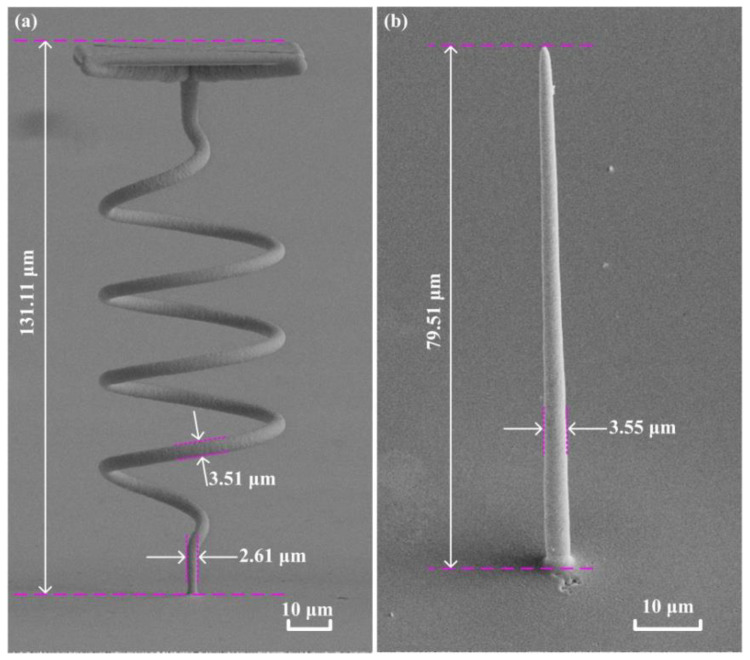
The high aspect ratio structures had been fabricated by localized electrochemical deposition and the aspect ratio is higher than 20. (**a**) a helix with flat surface. (**b**) a micro fine needle.

For the localized electrochemical micro-additive manufacturing of wire structures, it has been verified that the printing of microstructures can achieve a high aspect ratio during cylinders deposition. It is reported that the maximum aspect ratio can reach 400 [34]. It can also produce a high aspect ratio in the local manufacturing of the spiral structure with a flat top. The diameter of the deposit in Figure 2a is calculated according to 3.51 μm, and the aspect ratio can reach 37.4. Additionally, the aspect ratio of Figure 2b is 22.4. The maximum height that can be printed in the *Z*-axis direction is 70 mm. However, for thin linear structures, bending and dumping will occur. A single structure cannot exist upright and is prone to suffer print failure. The results of this experiment confirmed the ability of the high aspect ratio microstructure based on FluidFM. It is found in Figure 2b that the printed cylindrical structure has a thicker lower end and a thinner upper end. This is because the pressure is maintained at a constant rate during the printing process. Due to the diffusion of metal ions in the lower part of the electrodeposition body, there are some extra metal ions in the body solution. These metal ions continue to be subjected to secondary electroplating on the deposited surface, which results in a thicker lower end of the deposited linear structure. Additionally, the closer this plating position is to the local deposition region, the more obvious the phenomenon.

### 3.2. Volume Structure

**Figure 3:**
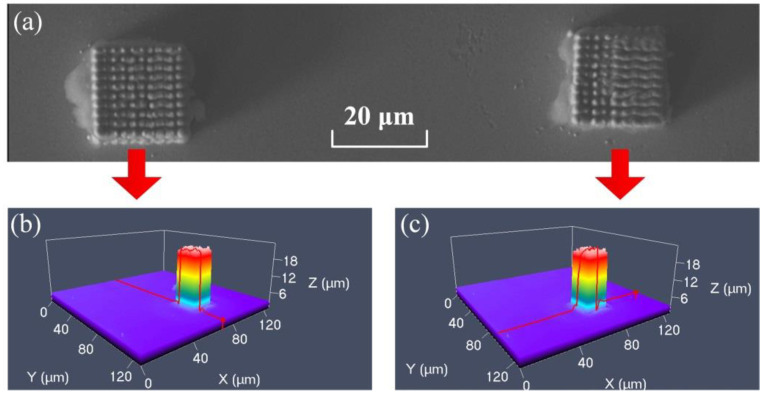
The two cubes were fabricated near each other. (**a**) The cube with a square surface and each boundary is 20 μm in length. (**b**,**c**) The dimension is measured by laser scanning confocal microscope for the first and second cube.

In order to verify the consistency and repeatability of the LECD process, two cubic microstructures with the same size and print parameters were fabricated simultaneously. From the perspective of the top view in Figure 3a, it can be observed that the top surfaces of the two cubes are almost the same. The two cubes in Figure 3a have a regular shape and high deposition quality. In Figure 3b, the dimensions are measured using a laser scanning confocal microscope. The verticality is relatively high between the side and the top surface of the deposit. The deposit height of the deposit is 20 μm, and almost no stray deposition occurs in the contact area between the deposit and the substrate. From the top surface of the electron microscope picture, the cubic structure is connected by 10 × 10 cylinders. Additionally, the diameter of each cylinder is about 2 μm. The mechanical properties of the materials need to be further verified.

**Figure 4:**
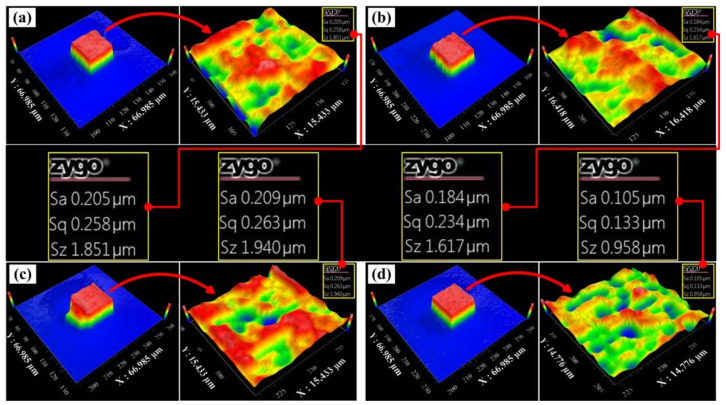
The roughness value was measured by a white light interferometer. Additionally, all the roughness value showed in four random cubes. (**a**) the roughness value of the first cube; (**b**) the roughness value of the second cube; (**c**) the roughness value of the third cube; (**d**) the roughness value of the fourth cube.

Four cubes were randomly selected on the working electrode substrate and the surface roughness value was measured. The area of the upper surface of the cube is 20 × 20 μm^2^, so it is difficult to measure with a contact surface roughness measuring instrument. In this study, the surface roughness of parts was measured using a white light interferometer. The measurement results showed that the surface roughness value of printed structures was between 0.1–0.2 μm. From the micro topography, the flatness of the upper surface was very high, but a few defects were observed on the verticality of the side and the top surface, as shown in Figure 4b. Figure 4c indicates that there are also a few defects on the front wall. It is because the gas pressure was unstable during the localized deposition process, causing more metal salt solution to flow out than expected. However, the strength of the electric field did not change, so the sidewalls did not deposit in a right way. The surface roughness values of the structures were measured separately, and the following results were obtained: the surface roughness Sa = 0.205 μm in Figure 4a, the surface roughness Sa = 0.184 μm in Figure 4b, and the surface roughness Sa = 0.209 μm in Figure 4c. Figure 4d shows the smallest surface roughness of Sa = 0.105 μm. Meanwhile, the deposition body has clear edges, flat surfaces and neat sides.

### 3.3. Complex Structure

**Figure 5:**
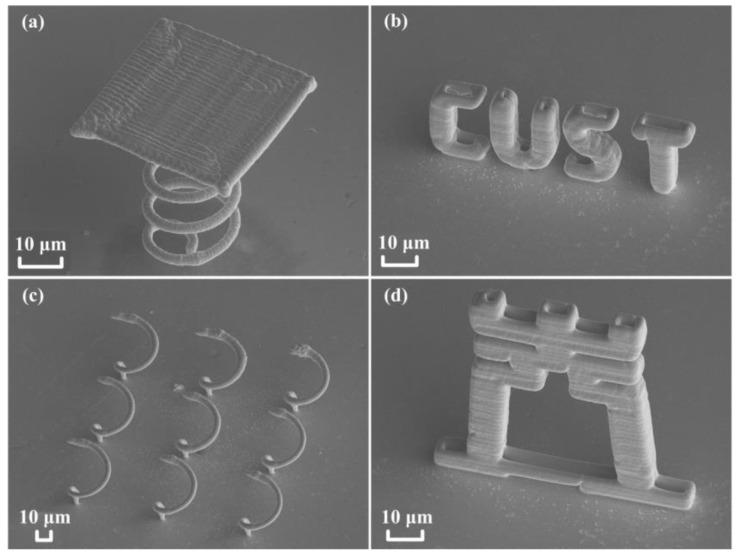
The complex microstructures precisely manufactured by electrochemical micro-additive manufacturing technology. (**a**) The structure with a flat on the top of lower spiral. (**b**) The abbreviation of the English alphabet for Changchun University of Science and Technology. (**c**) The 3 × 3 array structures of a half-circle spiral. (**d**) A Chinese character "light" shape.

Localized electrochemical micro-additive manufacturing technology with complex structures gives it a significant advantage. It can deposit many heterogeneous cantilever unsupported structures. In order to verify the excellent performance of the electrochemical additive manufacturing device, several cantilever structures printed free-form copper microstructures, as shown in Figure 5, which fully proves the superiority and feasibility of this additive manufacturing mode. A scanning electron microscope was employed to obtain the SEM image of the printed three-dimensional structure. The morphology and size of the printed copper metal three-dimensional part was observed. Figure 5a presents the flat top structure on the spiral under additive manufacturing. In the process of printing three-dimensional unsupported small and complex structures, the flat bottom structure with a spiral bottom is a typical unsupported structure. The area of the flat top structure above is much larger than the projected area of the bottom surface of the spiral support. A coil spring wire structure was used as the support structure, demonstrating the great advantage of the unsupported structure. Figure 5b shows the abbreviation of the English name of Changchun University of Science and Technology. It can be seen that all letters are of hollow structures. This technology can be freely switched between solid and hollow structure printing modes. Figure 5c indicates a 3 × 3 array structure of half-circle spiral. Figure 5d represents a shape of a Chinese character "light". It is indicated that each part of this structure is hollow. Figure 5d fully shows the superiority of this method in printing three-dimensional cantilever hollow structures and solid structures, and the feasibility of this highly localized electrochemical deposition fabrication is enhanced.

### 3.4. Big Area Structure

**Figure 6:**
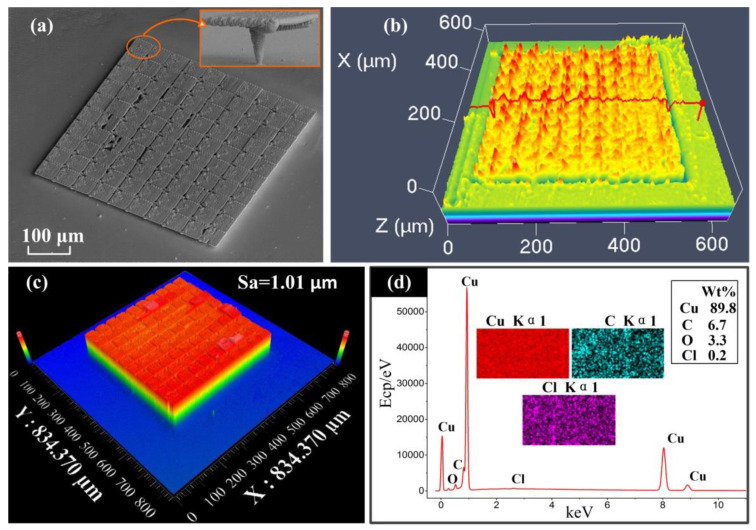
Production of large-area suspended surface. (**a**) SEM image of suspended planar array pattern, forming a 10 × 10 array structure, printing a large-area additive manufacturing surface of 400 μm × 400 μm, a single structure size of 40 μm × 40 μm. (**b**) Using laser scanning confocal microscopy to characterize the surface of the array pattern, protrusions appeared at the position where the small surface was spliced to the next surface, resulting in a very uneven surface. (**c**) The white light interferometer measures the macroscopic topography of the array plane. The top surface of the topography is suspended, and the upper surface is only a thin layer. So, the edges of the overall structure are neat. Additionally, the measured surface roughness value Sa = 1.01 μm. (**d**) The chemical element analysis by EDS based on the SEM. Additionally, the surface of the deposit contains elements of Cu, C, O, and Cl.

In order to verify the characteristics of FluidFM-based localized electrochemical deposition technology when printing large-area structures, we designed a large-area splicing structure. We arrayed the tiny structures in the upper right corner of Figure 6a to form 10 × 10 structures, and printed a 400 μm × 400 μm large-area thin planar cantilever structure. Figure 6b characterizes the convexity and flatness of the surface, using a laser confocal scanning microscope. It is found that the surface irregularity of the surface is significantly improved, as compared to the surface roughness of the body structure after a large-area deposition. As shown in Figure 6c, compared with the result of small surface deposition, the surface quality of the large surface is reduced and the surface roughness is significantly increased, reaching 1.01 μm. This article shows the composition analysis of the surface in the state of large-area printing through Figure 6d. The analysis result of surface scanning showed that 89.8% of the components were copper. Some oxides were also formed on the surface due to the presence of oxygen, and some carbon elements were also obtained during the test. This may be because it is a large-area print structure, and there is a tiny gap of 20 micrometers between the large-area structure and the substrate. There are indeed chemical elements in the deposition environment in the deposit, but except for the copper element, the mass fractions of other elements are less, and will inevitably interact with these during processing. The composition has little effect on the mechanical properties of the deposited structure. After printing, if the printed structure is protected with inert gas to prevent contact with air, it may reduce the content of C and O elements in the material, but it cannot be completely eliminated. This technique can be used to study the in-depth influence of surface microstructures on the bouncing properties of water droplets, and then reveal the hydrophilic and hydrophobic properties of the micro-topography of copper metal surfaces. The large-area metal micro-surfaces manufactured by this technology have important applications in active anti-reflection and anti-reflection performance.

### 3.5. Principles of Deposition

FluidFM technology-based metal LECD technology also has special features in the 3D structure microforming process, as shown in Figure S1 (Supporting Information). The principles of deposition structures formation covers the aspects of localized electric field formation, the precision liquid supply, and localized deposition completion. The model for reducing metal ions to atoms includes the following five steps: mass transfer, pre-conversion, charge transfer, atomic adsorption, and atomic crystallization. The main principles used are Fick’s law of diffusion, Nernst–Planck law, Navier–Stokes law, Butler–Volmer, Faraday’s law, and the volume growth of cylindrical deposits.

The electrochemical deposition model of electrochemical micro-additive manufacturing technology is realized by the diffusion, convection and electromigration of electrochemical species. In the traditional electrochemical deposition model, the convection of ions in a relatively static solution has less effect on the deposition process. However, the convection of ions plays a significant role in the LECD based on FluidFM. Due to pressure, convection occurs intermittently, and the volume of the metal salt solution flowed out each time varies depending on pressure. On the other hand, diffusion has also changed from a passive diffusion to an active one, and the scope of diffusion has become more complicated. The electromigration process of metal ions has not changed much in nature. The thickness of the electric double layer between the WE and the solution is a few tenths to a few nanometers. The gap between the WE of the FluidFM probe is about 250 nm. Therefore, the influence of the electric double layer is still inevitable during the highly localized deposition of the FluidFM probe.

**Figure 7:**
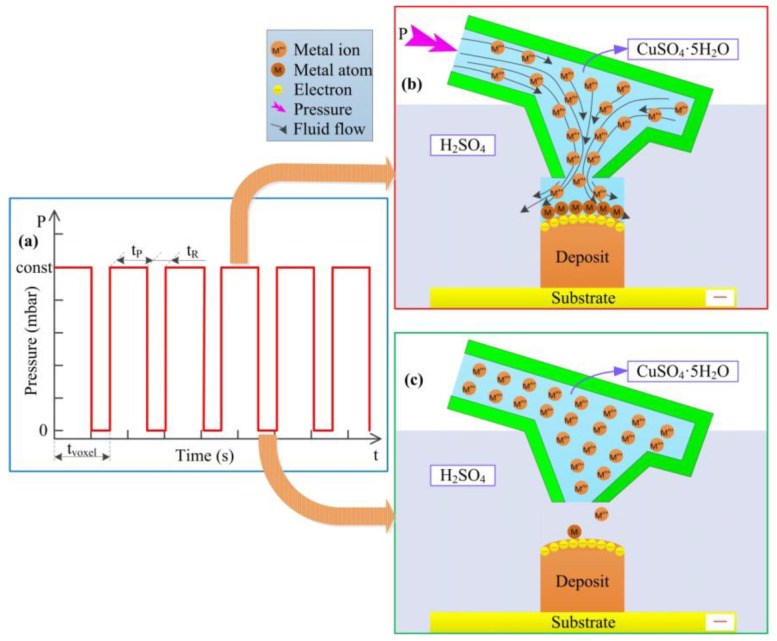
Theoretical diagram of the effect of pulse pressure on the deposition process. (**a**) is a relationship between the pressure and the time in process. (**b**) is the deposition process with pressure. (**c**) is the deposition process without pressure.

Figure 7a displays the pulse pressure in localized electrochemical deposition. As shown in Figure 7b, when pressure is applied (*t* = *t_P_*), *t_P_* is the time required to deposit a voxel. In the area far from the electric double layer, the volume of the fluid flow is controlled by the pressure *P*. *P* = const is set as a value in the program.

(1) $$\left\{\begin{array}{l} {\vec{J}}_{i}=-D_{i}\nabla c_{i}+z_{i}u_{i}Fc_{i}\nabla \phi_{i}+c_{i}\nu \\ \frac{\partial \vec{u}}{\partial t}+\left(\vec{u}\cdot \nabla \right)\vec{u}=-\frac{1}{\rho }\nabla P+g\\ \rho \cdot \nabla \vec{u}=0\\ P=const \end{array}\right\}\left(t=t_{P}\right)$$

As shown in Figure 7c, at the stage of the pressure disappears *t = t_R_*, *t_R_* is equal to the time it takes for the cantilever to return to the normal state after one voxel deposition, adding the time for the FluidFM probe head to rise to the initial inter electrode gap. The metal ion concentration *c_extra_* and the fluid velocity *u_tP_* during the *t_R_* time are both the remaining concentration and the final velocity at the end of the *t_P_* time.

(2) $$\left\{\begin{array}{l} {\vec{J}}_{i}=-D_{i}\nabla c_{i}+z_{i}u_{i}Fc_{i}\nabla \phi_{i}+c_{i}\nu \\ c_{i}=c_{extra}\\ \nu ={\vec{u}}_{t_{P}}\\ P=0 \end{array}\right\}\left(t=t_{R}\right)$$

where *D_i_* represents the rate of diffusion (m^2^/s); *c_i_* is the concentration gradient; ▽φ_i_ is the potential gradient; *z_i_* presents an ionic charge; *c_i_* is the concentration (mol/m^3^);and *F* is the faraday constant 96,500 (C/mol), *u_i_* is the mobility of the charged species, *ν* is the velocity vector of convective flow (m/s), *ρ* is the density (kg/m^3^), *u* is the velocity vector (m/s), *P* is the pressure which is a set value (mbar), *g* is the gravitational acceleration (m/s^2^). *t_P_* is the time of keeping the pressure open (s). *t_R_* is the time of keeping the pressure closed (s). *c_extra_* is the concentration of metal ions after deposition (mol/m^3^). *u_tP_* is the end speed of the metal ions flow (m/s).

The sum of *t_P_* and *t_R_* is the printing time of one deposit voxel. The effect of pulse voltage on the electrodeposition process has been studied in the previous literature. However, for the first time, the influence of external pulse pressure on deposition is proposed in this paper. The pulse pressure is achieved by controlling the pressure switch at the rear end of the FluidFM cantilever, to achieve quantitative and local deposition of the metal electrolyte at a certain time.

The current density and electric field distribution in the localized electrochemical deposition process are the key parameters that directly affect the deposition quality. These two parameters were closely monitored during the deposition process, and the polarization curve was used to detect the deposition quality during processing.

**Figure 8:**
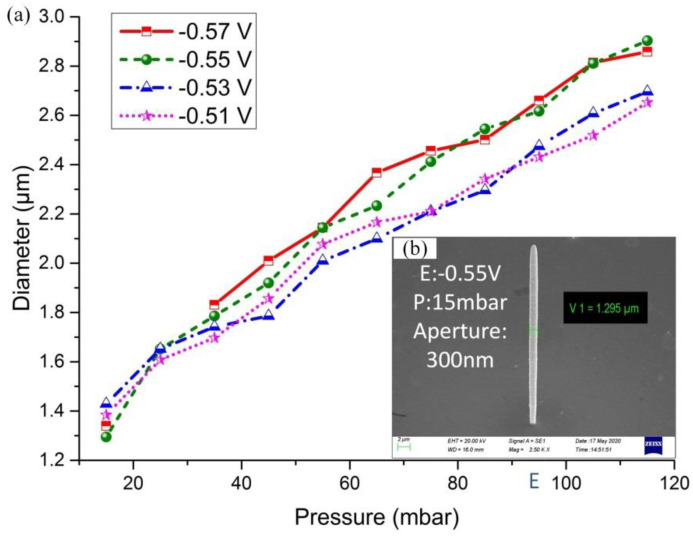
The diameters of the cylinder deposit with different pressures and voltages. (**a**) The graph of the data of diameter with different pressures. The diameter is measured by SEM picture. (**b**) The measurement line is marked on the deposit. Additionally, the diameter measured positioned on the half height of the cylinder deposit.

In order to more accurately measure the diameter of the printed cylinder in the SEM image, the array cylinder was crushed with tweezers on purpose, and the half-height diameter was measured respectively. The measurement positions were marked with lines, as shown in Figure 8b. The voltages from −0.51 V to −0.57 V were applied to the working electrode, and the scale of pressure was from 15 mbar to 115 mbar, at intervals of 10 mbar. The measured 12 diameter values are shown in Figure 8a for each voltage. The existence of the pressure gradient affects the convection efficiency of metal ions, determines the volume of electrolyte flowing out of the probe hole per second, and then determines the number of metal ions participating in the electrochemical reaction, and ultimately affects the volume of metal deposited per unit time.

## 4. Conclusions

This work focuses on the highly localized micro-additive manufacturing technology of electrochemical deposition based on FluidFM. This technology can directly fabricate metal structures with sub-micron to sub-millimeter dimension at sub-micron resolution. In this study, the problem of high locality is no longer achieved through micro-anode movement by ultra-high precision *Z*-axis motors. By uniformly arranging the electric field, the FluidFM probe was used to achieve the high localization of the electrolyte, which completed the highly localized electrochemical deposition manufacturing.

Firstly, the overall layout and design of the mechanical structure of the entire device were optimized. In order to meet the high localization requirements of electrochemical deposition, three moving axes were selected. Secondly, the process of electrochemical deposition of ions to atoms in a metal salt solution was described graphically. The mathematical model behind the 5 steps in the electrochemical process was analyzed, and the influence of key parameters of each step on the deposition process was investigated. The model for precipitating metal ions in metal solution to form metal atoms included the following five steps: mass transfer, pre-conversion, charge transfer, atomic adsorption, and atomic crystallization. Besides, a mathematical model for the material flux under the action of pulse pressure in a highly localized liquid feeding method was proposed in an innovative way. Finally, experimental verification was performed using a localized electrochemical deposition process, based on the FluidFM probe technology. The surface morphology and size measurements of the samples were tested using SEM and LSM, respectively, and the surface roughness of the samples was measured using a white light interferometer. The high aspect ratio, print consistency, surface roughness, and elemental analysis of the sediment were characterized from the four aspects of wire structure, volume structure, complex structure and big area structure. In order to verify the printing rate of this metal micro-additive manufacturing technology, a surface with an area of 400 μm × 400 μm was printed, which took 9.5 h. This technology has significant advantages in the forward fabrication of large-area microstructure surfaces, which can verify the hydrophilic and hydrophobic properties and anti-reflection properties of the surface.

For the subsequent exploration of this research, it is necessary to analyze the parameters of the metallographic structure of the forming structure, to find the influence of the deposition parameters on the microstructure of the sedimentary body. A series of studies on the mechanical properties and biological properties of microstructures deposited by the highly localized electrochemical deposition technology based on FluidFM are gradually carried out. This technology can be directly applied to the circuit space interconnection of nano-scale chips, manufacturing micro-sized sensors and common RF antennas and represents a great potential in a wide range of applications, creating great social and economic value.

## Figures and Tables

**Table 1:** Comparison of deposition effects in different technologies for metal micro structure.

Technology	Deposition Rate (μm/s)	Minimum Deposition Diameter	High Aspect Ratio	Complex Structure	Formation Quality of Surface	Environmental Requirements	Subsequent Processing
LECD-FluidFM	★★★	★★★★	★★★★★	★★★★★	★★★★★	★★★	No
LECD	★★★	★★★	★★★★	★★	★★★	★★★★	No
MCED	★★	★★★★★	★★★★	★★★★	★★★★	★★★★★	No
EcP	★★★	★★★★	★★★	★★★	★★★	★★★★	No
u-SLS	★★★★	★★★	★★★	★★★	★★★	★★★★★	Yes
FEBID	★	★★★★★	★★★	★★★★★	★★★★★	★★★★★	Yes
DIW	★★★★	★★	★★★	★★★	★★★★★	★★★★	Yes
MSL	★★★★★	★★★★	★★★★★	★★★★★	★★★★★	★★★★★	Yes
TPA	★★★	★★★★	★★★	★★★★★	★★★★★	★★★★★	Yes

The rows in Table 1 represent technology, and the columns represent technical characteristics. The number of stars represents how dependent the technology is on this parameter. ★★★★★ means that the technology is very dependent on this parameter, and ★ means that the technology is less dependent on this parameter.

**Table 2:** The parameters selected in the experimental validation.

Parameter	Value
Voltage (V)	−0.52 (Applied on the substrate)
Approaching voltage (mV)	50
Pressure (mbar)	20
Printing copper solution	CuSO_4_ (0.5 M); H_2_SO_4_ (51 mM); HCl (0.48 mM).
Supporting solution	H_2_SO_4_ (54 mM); HCl (0.5 mM)

## Supplementary Materials

The following are available online at https://www.mdpi.com/1996-1944/13/12/2783/s1, Figure S1: The diagram of the metal ions reduction from the solution to the solid WE surface. For the first step, CuSO_4_·5H_2_O is hydrolyzed to two kinds of the ions: [Cu·4H_2_O]^2+^ and [SO_4_ H_2_O]^2-^. Additionally, all the ions will transport according to convection, diffusion and electromigration mechanism. Next, the H_2_O of [Cu·4H_2_O]^2+^ is driven off leaving behind Cu^2+^. The third step, the Cu^2+^ ions get two electrons reducing to the Cu atoms. The forth step, the atoms are transported to the dislocation point of WE surface. The fifth step, the attached atoms go into the lattice convert to metal solids.
