# Supplementary material for: Modeling and Experimental Study of the Localized Electrochemical Micro Additive Manufacturing Technology Based on the FluidFM
Source: Materials (Basel). 2020 Jun 19;13(12):2783. doi: 10.3390/ma13122783 (PMC7345769; doi:10.3390/ma13122783)
Supplement: Supplementary file 1 [file materials-13-02783-s001.pdf]

materials

Supporting Information: Modeling and Experimental Study of the Localized Electrochemical Micro Additive Manufacturing Technology Based on the FluidFM

Wanfei Ren, Jinkai Xu *, Zhongxu Lian, Peng Yu and Huadong Yu *

Ministry of Education Key Laboratory for Cross-Scale Micro and Nano Manufacturing, Department of Mechanical and Electrical Engineering, Changchun University of Science and Technology, Changchun 130012, China; renwanfei2020@163.com (W.R.); lianzhongxv@126.com (Z.L.); 13620788904@163.com (P.Y.)
* Correspondence: xujinkai@cust.edu.cn (J.X.); yuhuadong@cust.edu.cn (H.Y.)

1. The Whole Mathematic model of the deposition process

Aiming to clearly demonstrate the forming mechanism of highly localized electrochemical deposition process, the section mathematic models were established after the deposition process. FluidFM technology-based metal LECD technology also has special features in the 3D structure microforming process. The mathematical model of 3D structure formation covers the aspects of localized electric field formation, the precision liquid supply, and localized deposition completion. The model for reducing metal ions to atoms includes the following five steps: mass transfer, pre-conversion, charge transfer, atomic adsorption, and atomic crystallization. The main principles used are Fick's law of diffusion, Nernst-Plank's law, Navier-Stokes' law, Butler-Volmer, Faraday's law, and volume growth of cylindrical deposits.

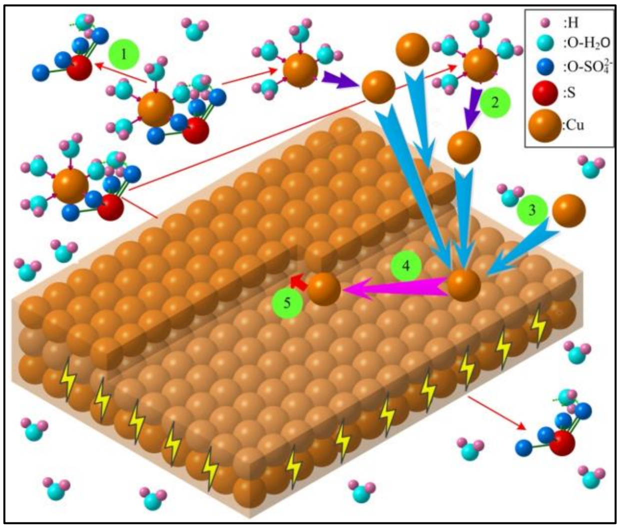

Figure S1. The diagram of the metal ions reduction from the solution to the solid WE surface. For the first step, $CuSO_4{\cdot}5H_2O$ is hydrolyzed to two kinds of the ions: $[Cu{\cdot}4H_2O]^{2+}$ and $[SO_4\ H_2O]^{2-}$. And all the ions will transport according to convection, diffusion and electromigration mechanism. Next, the H_2O of $[Cu{\cdot}4H_2O]^{2+}$ is driven off leaving behind Cu^{2+}. The third step, the Cu^{2+} ions get two electrons reducing to the Cu atoms. The forth step, the atoms are transported to the dislocation point of WE surface. The fifth step, the attached atoms go into the lattice convert to metal solids.

The electrochemical deposition model of electrochemical micro-additive manufacturing technology is realized by diffusion, convection and electromigration of electrochemical species. In the traditional electrochemical deposition model, the convection of ions in a relatively static solution has less effect on the deposition process. However, the convection of ions plays a significant role in the LECD based on FluidFM. Due to pressure, convection occurs intermittently, and the volume of

the metal salt solution flowed out each time varies depending on pressure. On the other hand, diffusion has also changed from passive diffusion to active one, and the scope of diffusion has become more complicated. The electromigration process of metal ions has not changed much in nature. The thickness of the electric double layer between the WE and the solution is a few tenths to a few nanometers. The gap between the WE of the FluidFM probe is about 250 nm. Therefore, the influence of the electric double layer is still inevitable during the highly localized deposition of the FluidFM probe.

In the FluidFM localized electrodeposition process, the metal salt solution is extruded from the channel of FluidFM cantilever under the action of air pressure, so it forms a positive ion diffusion state instead of spraying. The process of ions forming a metal solid is divided into five steps, and each step has its mathematical model. This work takes copper LECD as an example to illustrate the process of reducing copper ions to copper atoms. The localized liquid supply area was filled with copper sulfate solution. When the copper sulfate solution was added to the dilute sulfuric acid solution, the number of water molecules in the solution increased. As shown in Figure S1, the $CuSO_4{\cdot}5H_2O$ was hydrolyzed to two kinds of the ions: $[Cu{\cdot}4H_2O]^{2+}$ and $[SO_4\ H_2O]^{2-}$. When applying bias voltage between WE and CE in solution, all the ions were found to transport according to convection, diffusion and electromigration mechanism. When ions moved near the electric double layer, $[Cu{\cdot}4H_2O]^{2+}$could remove the H_2O and turn to Cu^{2+}. When the dehydrated copper ions moved closer to the WE surface, the Cu^{2+} ions reduced to the Cu atoms by getting two electrons. Reduced copper atoms adsorbed on the surface, and transported to the dislocation point of WE surface. After reaching the dislocation point, copper atoms would go into the lattice and convert to metal solids.

1.1. Mass Transfer

For general unstirred localized deposition, the convection has less effect on electrodeposition. For the localized electrodeposition process based on the FluidFM probe, the metal salt solution is derived from the cantilever of the FluidFM, so the electrochemical process at this time is an active localized electrodeposition process. In the range far from the deposition micro-region, the concentration of metal cations can be defaulted as 0. Under ideal conditions, all metal ions flowing from the tip of the AFM probe are reduced to metal atoms to form a solid. In this case, the microfluidic performance of the AFM probe microchannel is particularly important. Applying different air pressure values to the metal salt solution makes it possible to obtain the minimum size of the printed part online during the entire deposition process without changing the probe.

For the local electrochemical deposition process, it is still related to the deposition flux of the material and the relationship between the current density and the electric field distribution during the deposition process. Convection, diffusion and electromigration of metal ions participate in the deposition process. The three forms of ions transportation are determined by the gradient of pressure, concentration, and electric field strength, respectively. When pressure applied, the effect of convection is significant. Thus, the flux of species J_i corresponding to the diffusion is determined by the law of Nernst–Plank equation and Navier–Stokes equations.

The boundary condition, the ion current generated during the electrochemical process can be expressed by the Butler–Volmer equation:

$$\mathrm{i} = i_0\left[\exp\left(\frac{\alpha nF}{RT}\eta\right) - \exp\left(\frac{-\beta nF}{RT}\eta\right)\right];(\alpha + \beta = 1) \tag{1}$$

where R is the gas constant (8.31451 J/K mol), and T refers to the absolute temperature.

The current density and electric field distribution in the localized electrochemical deposition process are the key parameters that directly affect the deposition quality. These two parameters were closely monitored during the deposition process, and the polarization curve was used to detect the deposition quality during processing.

1.2. Pre-Conversion and Charge Transfer

The reaction particles that have migrated near the electrode surface undergo a chemical conversion reaction, and metal hydrated ions rearrange on the electrode surface.

$$M^{n+} \cdot m\ H_2O \leftrightarrow M^{n+} + m\ H_2O \quad (2)$$

The metal ions are reacted to metal atoms via obtaining several electrons. LECD process is an emerging technology based on ultra-precision electroplating and electroforming. The technology reduces metal ions in the electrolyte solution to metal atoms near the cathode surface which enter the metal lattice to form a deposition situ self-assembly. The principle of the chemical reduction reaction is mainly as follows:

$$M^{n+} + ne \rightarrow M\ (s) \quad (3)$$

Compared with the deposition of metal ions in the cathode, the anode of the electrolytic cell undergoes an oxidation reaction, which corresponds to the evolution of oxygen in the aqueous solution. The oxygen will flow out of the electrolyte tank along the edge of the carbon anode. The reaction on the anode is:

$$\frac{n}{2} H_2O - ne \rightarrow \frac{n}{4} O_2 \uparrow + nH^+ \quad (4)$$

In the LECD process based on FluidFM, the working electrode and the counter electrode were placed far away. The solid deposited on the working electrode generated oxygen on the counter electrode, and a large amount of solution was separated in the distance between the oxygen and the deposited body. Due to the adsorption of carbon, oxygen was adsorbed on the surface of the carbon anode to avoid oxidation of metal solids in the deposit.

1.3. Atomic Adsorption and Crystallization

The LECD process based on the FluidFM probe still needs to follow Faraday's law of electrolysis. The quality of the formation and growth of the precipitated solid is directly proportional to the amount of electricity passing through the electrode. Newly adsorbed metal atoms diffused along the electrode surface to the growth point and entered the metal lattice to grow, or gathered with other new atoms to form crystal nuclei and grew, thereby forming crystals. Nucleation and spiral dislocations grew during the crystallization process.

According to Faraday's law, the mass of a chemically changed substance at the electrode interface is proportional to the amount of electricity passed in.

$$m = \frac{M \times I \times t}{F \times n} \quad (5)$$

where M presents molar mass, I is the current intensity, n denotes the valence, and t is the deposition time.

In view of the fact that the smallest voxels of the deposition structure are spherical cylinders, the cross-sectional area of the deposit can be treated as a circle shape. The mass is equal to the product of the cross-sectional area and the height and density of the sediment, where the diameter and height of the deposit can be measured by SEM figures.

$$\pi R^2 \times h \times \rho_{Cu} = \frac{M \times I \times t}{F \times n} \quad (6)$$

where ρ_{Cu} is the density of copper.

© 2020 by the authors. Submitted for possible open access publication under the terms and conditions of the Creative Commons Attribution (CC BY) license (http://creativecommons.org/licenses/by/4.0/).
